# Cross-Over Study Evaluating Photobiomodulation in Pain Control in the Lower Limb

**DOI:** 10.7759/cureus.64914

**Published:** 2024-07-19

**Authors:** Lívia Maria Pereira de Godoy, Henrique Jose Pereira de Godoy, Jose Maria Pereira de Godoy

**Affiliations:** 1 Dermatology, Instituto Lauro Souza de Lima-Bauru-Brazil, São José do Rio Preto, BRA; 2 Research, Clínica Godoy, São José do Rio Preto, BRA; 3 Vascular Surgery, Faculdade de Mediocina de Sao Jose do Rio Preto (FAMERP), São José do Rio Preto, BRA; 4 General Practice, Clínica Godoy, São José do Rio Preto, BRA; 5 Cardiology and Cardiovascular Surgery, Faculdade de Mediocina de Sao Jose do Rio Preto (FAMERP), São José do Rio Preto, BRA; 6 Angiology and Vascular Surgery, Clínica Godoy, São José do Rio Preto, BRA

**Keywords:** laser, treatment, pain, chronic, leg ulcer

## Abstract

Introduction

Photobiomodulation is an emerging treatment modality in dermatology, with increasing use in doctors’ offices. Photobiomodulation is the use of various light sources in the red light (620-700 nm) and near-infrared (700-1440 nm) spectrums as a form of light therapy.

Objective

The objective of the present study was to evaluate the use of photobiomodulation to improve pain in patients who take analgesics daily for chronic non-arterial leg ulcers.

Method

A cohort trial was performed with 20 patients to evaluate the improvement in pain, patient tolerance to treatment, and evolution of chronic, difficult-to-heal leg ulcers treated with low-frequency laser. Data were entered into a Microsoft Excel spreadsheet (Microsoft Corporation, Redmond, WA, US). Statistical analysis used the Stats Direct 3 program with significance being set at an alpha error of 5% (p-value <0.05).

Results

An improvement in pain immediately after the first session was experienced by 18/20 patients; these patients remained pain-free for at least four days. One patient had suffered much pain during the entire day; a biopsy with culture and antibiogram was performed with the pain improving after treating an infection with appropriate antibiotics. However, one other case reported no improvement in the pain.

Conclusion

Photobiomodulation is an optional adjuvant therapy to improve pain in the treatment of chronic, difficult-to-heal leg ulcers.

## Introduction

Venous leg ulcers are caused by impaired flow of venous and lymphatic fluids from the lower leg toward the central circulatory system. Affecting 3% to 5% of over-65-year-old people, they are one of the most common types of chronic wounds [1]. Assessing the stage and severity of chronic diabetic foot ulcers is vital to increasing the cure rate and selecting appropriate treatment [2].

Photobiomodulation (PBM) is an emerging treatment modality in dermatology with increasing use in doctor’s offices. PBM is the use of light sources in the red light (620-700 nm) and near-infrared (700-1440 nm) spectrum as a form of light therapy [3]. Systematic reviews report that PBM presents minimal risk and offers promising improvements in forming granulation tissue, wound healing duration, and degree of pain in diabetic patients [4-6]. Low-dose photodynamic therapy (LDPDT) positively impacts wound healing in diabetic rat and mouse models [7,8]. Additionally, a meta-analysis reports significantly greater reductions in ulcer size and complete healing rate in patients with diabetic foot as compared to control individuals [9].

Another systematic review states that PBM alone or combined with other therapies can moderate the inflammatory response and accelerate the wound-healing process in preclinical models of diabetic wounds. However, few studies have evaluated, in detail, the functions of polarized macrophages and M2 subtypes in wound healing [10]. A randomized trial reports that low-level laser light therapy reduces tissue regeneration time, contributing to advances in wound treatment [11]. Blue light treatment, in addition to standard treatment, consistently accelerates the rate of the re-epithelialization of chronic wounds, especially venous leg ulcers, and increases the chances of complete wound healing within 10 weeks [12].

Another study shows that low-frequency lasers can be useful in the treatment of lower limb ulcers but there is a need to determine the specific parameters of each type of laser and the therapeutic responses to each type of laser. The objective of the present study was to evaluate the use of PBM to improve pain in patients who take analgesics daily for chronic non-arterial leg ulcers.

## Materials and methods

A prospective cross-over study was performed to evaluate improvements in pain, tolerance to therapy, and healing of chronic, difficult-to-heal leg ulcers treated with PBM. Twenty consecutive patients with painful chronic ulcers of the lower limbs that were not improving with conventional management were enrolled in this study between 2022 and 2023 at Hospital de Base of the Medical School in São José do Rio Preto-Brazil and Clínica Godoy-Sao Jose do Rio Preto-Brazil. Patient information included age and sex.

Inclusion criteria

Patients with venous lower limb ulcers less than or equal to 5 cm in diameter, healable wounds, non-cancerous wounds, and daily pain needing continuous analgesics were included in the study.

Exclusion criteria

Pregnant or breast-feeding women, participants taking photosensitive drugs for concomitant disease, and patients with arterial ulcers were excluded from the study.

Treatment procedure

All patients were being treated for venous ulcers, with these treatments being maintained during this study even though conventional therapy was unsuccessful in resolving the lesion. For the PBM treatment, patients were instructed to remain in a comfortable position. Once local asepsis was performed, low-frequency laser sessions using a 3-joule red laser (Ecco Reability, Ecco Fibras, Campinas, Brazil) with a power of 660 mW and duration of 25 seconds at four-day intervals were added as adjuvant therapy. A visual analog scale (VAS) [13] unidimensional measure of pain intensity for pain was used in a straight horizontal line of fixed length, providing a range of scores from 0-10, normative scale no pain (0), mild pain (1-3), moderate pain (>3-7), and severe pain (>7-10) and was completed before the first session and then on returning to the clinic after four days.

Statistical analysis

Data were entered into a Microsoft Excel spreadsheet (Microsoft Corporation, Redmond, WA, US) and are reported as descriptive statistics. The Mann-Whitney test in the StatsDirect software (version 3; https://www.statsdirect.com/) was used for statistical analysis with significance set for an alpha error of 5% (p-value <0.05).

Ethical approval

The study was approved by the Ethical Committee of Faculdade de Medicina de Sao Jose do Rio Preto #4.161.679, CAAE 30596920.0.0000.5415. The patients signed the consent form.

## Results

 This study enrolled 16 female and 4 male patients aged between 45 and 72 years old with a mean age of 61 (standard deviation: 10.3) years. Of these, 10 reported that the lesions appeared spontaneously, 5 after trauma, and 5 were diabetic, but none had any evidence of ischemia. In the results obtained with the VAS instrument, 18 patients had an immediate improvement in pain after the first laser session with these patients remaining pain-free for at least four days. One had suffered much pain the entire day; a biopsy with culture and antibiogram was performed with the pain improving after treating an infection with appropriate antibiotics. However, one other case reported no improvement in the pain. Table 1 shows the results of the pain analog scale. A significant difference was detected between the before and after evaluated by the Mann-Whitney U test p-value < 0.0001.

**Table 1:** Pain analog scale results before and four days after the low-frequency laser session

Patient #			Pain analog scale score	
	Sex	Age	Before	After
1	woman	48	6	2
2	woman	72	5	1
3	woman	48	4	1
4	woman	69	7	2
5	man	70	6	2
6	man	67	5	2
7	woman	69	9	9
8	woman	63	3	2
9	woman	67	5	3
10	man	48	8	3
11	woman	47	6	3
12	woman	50	5	3
13	woman	71	5	3
14	woman	68	4	4
15	man	51	7	3
16	woman	70	7	5
17	woman	58	6	4
18	woman	68	5	3
19	woman	45	8	5
20	woman	53	6	3

**Figure 1:**
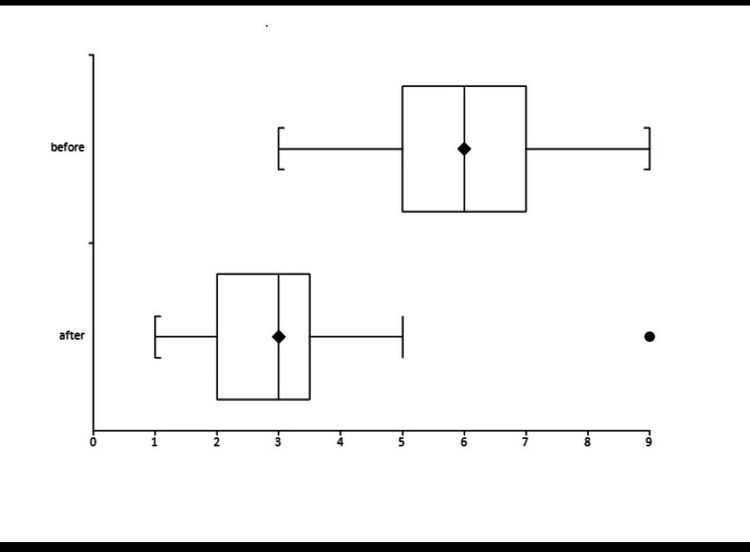
Comparing (minimum, median, and maximum) analog pain scale scores before and four days after the first laser session

Table 1 shows the results of the pain analog scale before and after the application of the therapeutic. Figure 1 compares analog pain scale scores (minimum, median, and maximum) before and four days after the first laser session.

## Discussion

The present study shows that PBM is an adjuvant option in the treatment of chronic painful ulcers resistant to conventional treatment. An improvement in pain, even in patients who took analgesics daily, was detected from the first laser session as most patients reported that they reduced or even stopped taking painkillers and that they observed that the wound began to heal. Clinical analyses confirm this observation, however, the time the lesions took to heal was not documented. The first patient who received PBM in our department had had an ulcerated lesion for six months. The patient reported that it was very painful during this period and the pain improved from the first laser session with the lesion healing within about one month, a fact that inspired the current research [5].

The use of light as a therapy dates to ancient civilizations, with ancient Egyptians and Indians making use of sunlight (heliotherapy) for healing and promoting health [14]. The precise mechanisms of action of PBM are not fully known and understood; however, a broad range of effects at the molecular, cellular, and tissular levels have been observed [15].

The analgesic action induced by PBM can be explained by the modulation of inflammatory mediators. The release of beta-endorphin tends to limit the excitability of painful receptors and eliminate pain sources [16]. Studies about analgesic properties of PBM, beyond the resolution of cortical coherence and brain wave pattern disruptions, are supported by a wealth of data that provide insight into the possible delivery of pre-emptive PBM in the prevention and development of persistent pain [17].

The literature shows that PBM is a safe and effective option, but some reports do not corroborate these conclusions [2-6]. Thus, there is a need to standardize the treatment method, including reporting the type of laser and specific types of wounds such as diabetic foot and venous and traumatic ulcers. In the present study, the patients were selected consecutively so the etiologies of the lesions were not taken into account; the results were positive in 90% of cases.

One of the patients, whose healing did not improve, had a chronic venous ulcer with intense fibrosis. It is suggested that each pathophysiological aspect of ulcerated lesions should be analyzed in order to indicate the best therapeutic option. Venous ulcers, for example, respond well to treatments using Unna boots [12]. Even so, the search for new alternatives is necessary. In a pilot study (in press), we found that high-frequency laser is another possible adjuvant therapy [3].

Several options have been described to improve the healing of chronic wounds of the lower limbs, however, it is important to consider the cost of management and standardization of the different methods. Cohort studies to evaluate the healing time using conventional treatment associated with PBM as an adjuvant therapy for each type of wound would be enlightening. The limitations of the study relate to the type of study, which was a cross-over and ideally a clinical trial. Other aspects, such as wound healing time and the need for antibiotics are important data to be evaluated. However, the assessment of pain in this study already provides important information and justifies its use as an adjuvant in treatment.

## Conclusions

Photobiomodulation as a non-invasive, low-risk modality can deliver significant improvements in the healing of leg ulcers after therapy. Is an optional adjuvant therapy to improve pain in the treatment of chronic, difficult-to-heal leg ulcers with significant pain. Despite all the positive effects of PBM on painful conditions, it is important to keep in mind that the PBM parameters used are crucial for the success of the therapy.
